# A Randomized, Investigator‐Blinded, Split‐Face, Controlled Trial Evaluating the Efficacy and Satisfaction of a Topical Product Containing Blueberry Extract and Pro‐Xylane Combined With Micro‐Focused Ultrasound for Anti‐aging

**DOI:** 10.1111/jocd.70281

**Published:** 2025-07-10

**Authors:** Xiaoling Jiang, Shiqi Peng, Ying Chen, Yuqing Nan, Weimin Song

**Affiliations:** ^1^ Hang Zhou YesSkin Hospital Hangzhou Zhejiang China

**Keywords:** blueberry extract, integrated skincare, micro‐focused ultrasound, pro‐xylane, skin aging

## Abstract

**Background:**

Micro‐focused ultrasound (MFUS) has been effective in treating skin aging, which manifests as skin thinning, reduced elasticity, wrinkling, etc.

**Aims:**

This study aims to evaluate the efficacy and satisfaction of combining a product containing blueberry extract and Pro‐xylane (A.G.E. cream) with MFUS in antiaging effects in the Chinese population.

**Methods:**

In this randomized, investigator‐blinded, controlled trial, patients aged 18–65 with skin roughness, sagging, laxity, or fine lines/wrinkles were enrolled. After MFUS, one side of patients' faces was randomly assigned to apply the A.G.E. cream and the other side with the standard moisturizer. Patients were followed up for 180 days. The primary endpoint was the Global Aesthetic Improvement Scale (GAIS). Secondary endpoints included percentage changes from baseline in fine lines, skin elasticity, dermal thickness, and transepidermal water loss. Patient and dermatologist satisfaction was evaluated using a questionnaire.

**Results:**

A total of 50 patients were included in the study, with 49 (98%) being female. The mean ± SD age was 33.7 ± 6.4 years. For the primary endpoint, the intervention side demonstrated significant improvement in GAIS compared to the control side on Day 90 (*p* < 0.001). For secondary endpoints, the A.G.E. cream use after MFUS significantly improved skin elasticity at the cheeks and mouth corners on multiple follow‐up days and improved fine lines at the mouth corners on Day 180. Patients and dermatologists reported higher satisfaction levels with the aesthetic outcomes of the intervention side.

**Conclusions:**

The application of the A.G.E. cream following MFUS treatment improved skin elasticity, dermal thickness, fine lines, and patient/dermatologist satisfaction.

**Trial Registration:**

ClinicalTrials.gov identifier: NCT05748470

## 1 Introduction

Skin aging is a multifaceted process that involves both intrinsic and extrinsic aging, manifesting as thinning of the skin, reduced elasticity, increased fragility, and visible phenotypes such as wrinkling, rough texture, dyspigmentation, and so forth. The clinical features of photoaging in Asian skin, such as changes in pigmentation and the appearance of wrinkles, differ from those observed in Caucasian skin [1]. A previous study has demonstrated that wrinkle onset is delayed by approximately 10 years in Chinese women as compared to French women [2]. In an analysis to characterize the trajectory of skin aging in Chinese women, it was found that ages 32 and 58 showed the largest number of differentially changed aging phenotypes, representing rapid skin aging at these age periods [3].

To combat photoaging and the consequent wrinkles and laxity, focused ultrasound therapy (FUS), specifically micro‐focused ultrasound (MFUS), has emerged as an effective and overall safe noninvasive treatment option through its skin tightening and rejuvenation effects [4, 5]. MFUS utilizes high‐energy waves to target specific focal points on subcutaneous tissue and creates a thermal injury zone in the mid‐to‐deep reticular layers of the dermis and subdermis while preserving the integrity of papillary dermal and epidermal layers [6]. The heat produced during the process at the thermal coagulation points leads to collagen denaturation, and thereafter the wound healing process is triggered, leading to collagen remodeling and dermal thickening through inflammatory mediators [7]. In addition to antiaging effects, MFUS has also paved its way into various applications in dermatology, such as reducing enlarged facial pores [8] and treatment of hyperhidrosis [9].

Accumulating evidence has suggested a synergistic effect in integrating aesthetic procedures and post‐procedure skincare regimens to prevent complications, promote recovery, and enhance patient satisfaction [10, 11, 12]. Previous studies have explored the combination of MFUS with other cosmetic procedures such as intradermal incobotulinumtoxin‐A [13] and dilute calcium hydroxylapatite filler [14]. However, there remains a paucity of clinical evidence regarding the synergy of MFUS with other functional topical agents for antiaging. A.G.E. Interpreter Advanced Anti‐wrinkle Cream (A.G.E. cream). Blueberry extract, the cream's key active ingredient, is rich in micronutrients such as vitamin E and C, and polyphenolic compounds, known for their antioxidant and anti‐inflammatory properties [15, 16, 17]. Previous studies on its direct topical application have shown that blueberry extract can protect the skin from damage induced by ultraviolet radiation and ozone exposure while also reducing collagen degradation, thereby contributing to its antiaging benefits [18, 19, 20, 21]. Additionally, prior clinical trials also illustrated that products containing blueberry extract and Pro‐xylene had promising results in reducing signs of aging like wrinkles and fine lines [22, 23]. Therefore, it is hypothesized that incorporating A.G.E. cream into the treatment regimen may amplify and extend the antiaging benefits of MFUS. This study aims to evaluate the efficacy and satisfaction levels of both patients and dermatologists in using a combination of MFUS and A.G.E. cream to address facial aging concerns in the Chinese population.

## 2 Materials and Methods

### 2.1 Study Design and Patients

This study is a randomized, investigator‐blinded, split‐face, controlled trial conducted at the YESSKIN Clinic in Hangzhou, China.

Patients aged between 18 and 65 with facial skin roughness, sagging, laxity, or fine lines/wrinkles were eligible to participate in the trial. Patients were excluded if meeting any of the following criteria: (1) patient who was contraindicated in ultrasound therapy (e.g., malignancy, systemic infections, severe heart diseases, bleeding tendencies, etc.); (2) patient who was allergic to any ingredients in the A.G.E. cream; (3) patient who had existed hypertrophic scars or prone to keloids.

Informed consents were obtained from all patients before the enrollment. This trial was approved by the Ethics Committee of YESSKIN Clinic.

### 2.2 Randomization and Blinding

After receiving the MFUS treatment, patients were randomized to designate their left or right side of the face as the intervention side and the other side as the control side in a 1:1 ratio based on a pre‐determined random number table. Independent investigators were blinded when evaluating clinical endpoints.

### 2.3 Interventions and Follow‐Up

**FIGURE 1:**
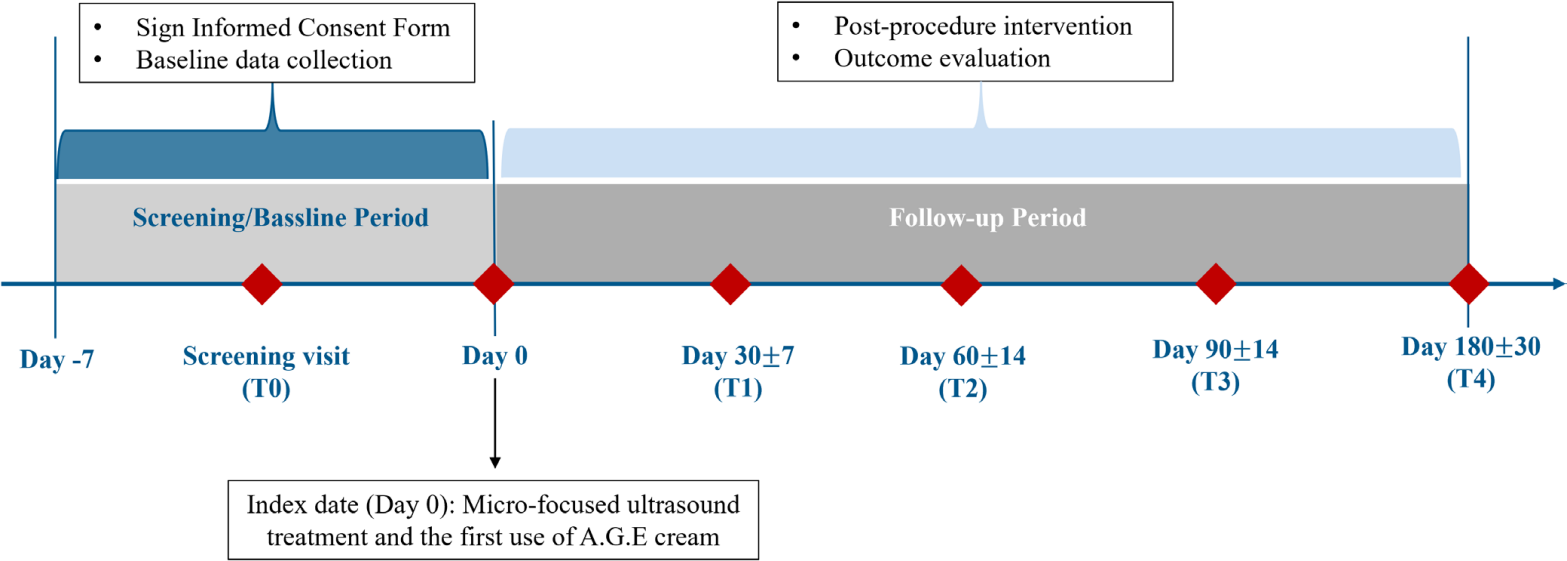
Schematic of study design.

This study consisted of a total of 6 visits, including the screening visit (T0: day −7 to 0), enrollment and treatment visit, 4 follow‐up visits on days 30 ± 7 (T1), 60 ± 14 (T2), 90 ± 14 (T3), and 180 ± 30 (T4) after MFUS treatment (Figure 1).

All patients enrolled in the study received the MFUS treatment on both sides of their faces (Device: MFUS One; Forehead: Depth 3 mm, 3.76–6.63 W, 10 Hz; Cheeks: Depth 4.5 mm, 3.76–6.63 W, 10 Hz). During the 180‐day follow‐up period after MFUS treatment, patients were instructed to apply a topical product containing blueberry extract and Pro‐xylane (A.G.E. Interrupter Cream, SkinCeuticals Inc.) to the intervention side, while a standard facial moisturizer with the key ingredient of glycerin was applied to the control side. Patients were instructed to apply the topical product or standard moisturizer twice daily, both in the morning and at night. A.G.E. cream contains 4% blueberry extract, 30% Pro‐xylane (C‐xyloside), and 0.2% salicyloyl‐phytosphingosine. During the study, the researchers set regular reminders to follow up with patients, and patients were required to keep a daily diary of their product applications. Additionally, at each follow‐up visit, researchers provided patient education to ensure patient adherence. Throughout the study, patients were recommended to adopt protective measures against ultraviolet exposure, such as applying sunscreen and wearing protective gear like hats or facial coverings.

### 2.4 Endpoints and Assessments

In this study, the primary endpoint was the Global Aesthetic Improvement Scale (GAIS) (1: worse; 2: no change; 3: improved; 4: much improved; 5: very much improved), visually assessed by independent investigators on day 90 after the procedure. Secondary endpoints included the change from baseline in fine lines, the percentage changes from baseline in skin elasticity, dermal thickness, transepidermal water loss (TEWL) on day 30, day 60, day 90, and day 180, as well as satisfaction levels of both patients and dermatologists. Fine lines were assessed using Griffith's scale. Skin elasticity, dermal thickness, and TEWL were measured using the DermaLab Combo system (Cortex Technology). Fine lines, skin elasticity, dermal thickness, and TEWL were evaluated at the forehead, cheeks, and mouth corner for each side. The assessment of patient and dermatologist satisfaction was conducted through a questionnaire that encompassed five key aspects: overall aesthetic outcome, wrinkle improvement, skin elasticity improvement, skin plumpness improvement, and skin hydration improvement.

Any adverse events (AEs) or serious adverse events (SAEs) would be recorded throughout the study.

### 2.5 Statistical Analysis

Upon recruiting 50 patients, and considering a standard deviation of 0.25, a significance level of 0.05, and a dropout rate of 20%, the study is powered at 80% to detect a difference of 0.15 for GAIS on day 90 [24].

Paired *t*‐tests or Wilcoxon signed‐rank tests were used in endpoint analysis. The significance level of < 0.05 was applied to all tests. All analyses were conducted using R (version 3.4.1).

## 3 Results

### 3.1 Baseline Characteristics

A total of 50 patients were included in this study, with 49 of them being female (98%). The mean ± SD age was 33.7 ± 6.4 years. There was no statistically significant difference in skin characteristics between the intervention and the control side at baseline.

### 3.2 Primary Endpoint

The study found that the intervention side that applied A.G.E. cream demonstrated significant improvements in aesthetic outcomes compared to the control side on Day 90 (*p* < 0.001) and Day 180 (*p* = 0.020) regarding GAIS evaluated by investigators. Specifically, a higher percentage of patients were rated as “Much improved” or “Very much improved” at the intervention side compared to the control side on day 90 (“Much improved”: 46.94% vs. 38.78%; “Very much improved”: 36.73% vs. 12.24%) and day 180 (“Very much improved”: 20.83% vs. 8.33%). (Table 1).

**TABLE 1:** Global Aesthetic Improvement Scale during follow‐up.

Follow‐up visit	GAIS	Intervention (A.G.E. cream)	Control (standard moisturizer)	*p* ^a^
T1: Day 30	Worsened	0 (0%)	0 (0%)	0.832
	No change	0 (0%)	1 (2.00%)	
	Improved	12 (24.00%)	11 (22.00%)	
	Much improved	33 (66.00%)	33 (66.00%)	
	Very much improved	5 (10.00%)	5 (10.00%)	
T2: Day 60	Worsened	0 (0%)	0 (0%)	0.842
	No change	0 (0%)	1 (2.04%)	
	Improved	14 (28.57%)	12 (24.49%)	
	Much improved	20 (40.82%)	23 (46.94%)	
	Very much improved	15 (30.61%)	13 (26.53%)	
T3: Day 90	Worsened	0 (0%)	0 (0%)	< 0.001***
	No change	0 (0%)	0 (0%)	
	Improved	8 (16.33%)	24 (48.98%)	
	Much improved	23 (46.94%)	19 (38.78%)	
	Very much improved	18 (36.73%)	6 (12.24%)	
T4: Day 180	Worsened	0 (0%)	0 (0%)	0.020*
	No change	0 (0%)	0 (0%)	
	Improved	9 (18.75%)	13 (27.08%)	
	Much improved	29 (60.42%)	31 (64.58%)	
	Very much improved	10 (20.83%)	4 (8.33%)	

Abbreviation: GAIS, Global Aesthetic Improvement Scale.

^a^ *p* Values were derived from Wilcoxon signed‐rank tests.

* *p* < 0.05.

*** *p* < 0.001.

### 3.3 Secondary Endpoints

**FIGURE 2:**
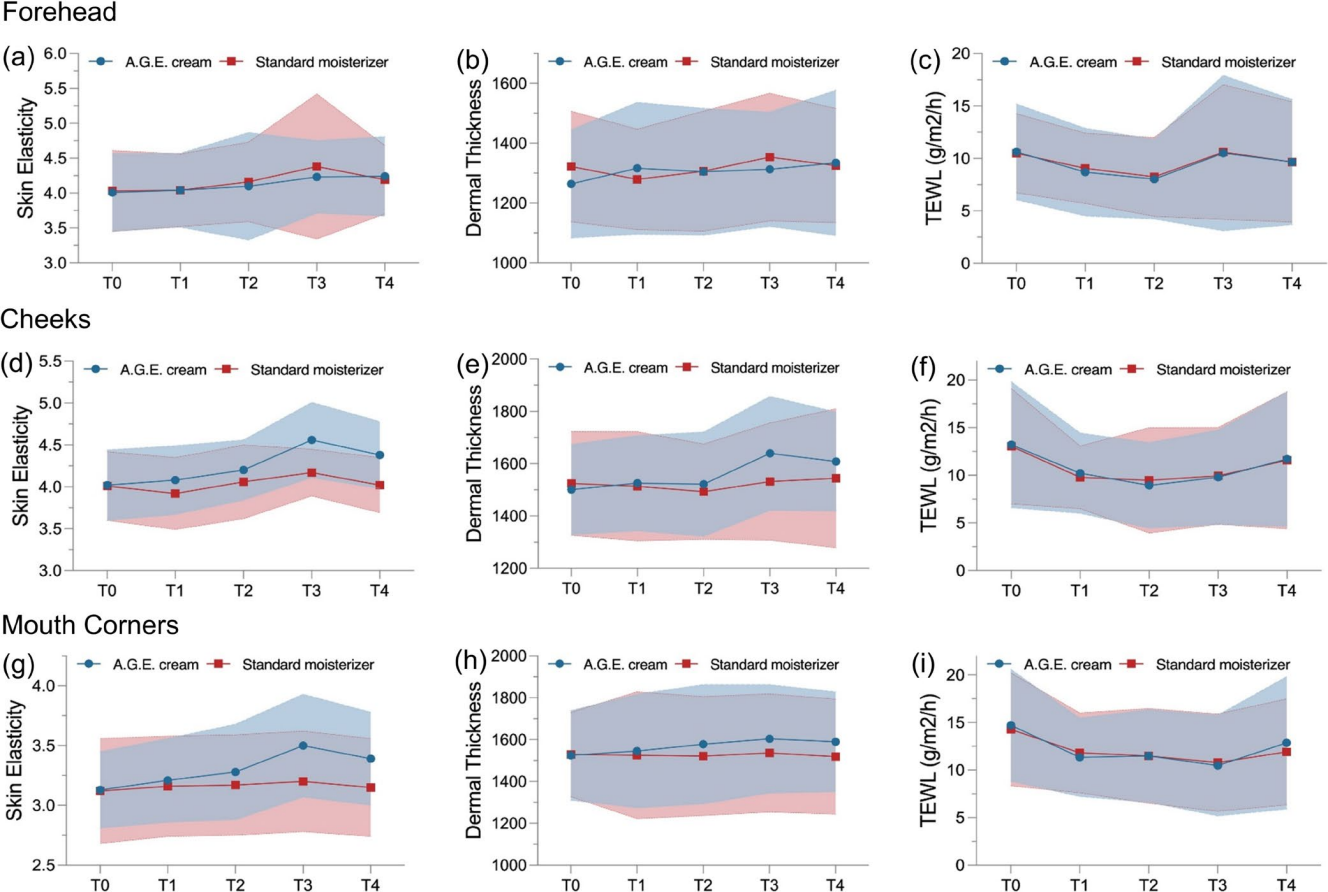
Changes in skin parameters throughout the study at forehead, cheeks, and mouth corners. (a) Skin elasticity at forehead; (b) Dermal thickness at forehead; (c) TEWL at forehead; (d) Skin elasticity at cheeks; (e) Dermal thickness at cheeks; (f) TEWL at cheeks; (g) Skin elasticity at mouth corners; (h) Dermal thickness at mouth corners; (i) TEWL at mouth corners; T0: Baseline; T1: Day 30; T2: Day 60; T3: Day 90; T4: Day 180; TEWL, transepidermal water loss. The blue‐ and red‐shaded areas represent the ranges between the standard deviation added and subtracted from the mean value in A.G.E. cream and standard moisturizer side, respectively.

This study found that the A.G.E. cream‐applied side showed higher skin elasticity than the control side regardless of regions of the face. (Figure 2a,d,g) Statistically, A.G.E. creams made significantly greater improvement on skin elasticity at cheeks on day 30 (Δ = 3.99, *p* < 0.001), day 60 (Δ = 3.55, *p* = 0.006), day 90 (Δ = 9.36, *p* < 0.001), and day 180 (Δ = 8.65, *p* < 0.001), and at mouth corners on day 90 (Δ = 8.73, *p* = 0.001) and day 180 (Δ = 6.12, *p* = 0.005) after MFUS, compared to the standard moisturizer (Table 2).

**TABLE 2:** Percentage changes from baseline in skin parameters at both sides during follow‐up.

Skin parameters	Follow‐up time points	*N*	Mean percentage change from baseline, mean ± SD		Difference between groups (Δ), mean ± SD	*p* ^a^
			Intervention	Control		
Skin elasticity						
Forehead	T1: Day 30	49	1.67 ± 13.60	1.36 ± 14.62	0.31 ± 17.43	0.9
	T2: Day 60	47	3.41 ± 20.12	4.18 ± 15.14	−0.62 ± 21.32	0.842
	T3: Day 90	48	6.42 ± 12.58	10.11 ± 30.50	−3.68 ± 27.86	0.843
	T4: Day 180	47	6.46 ± 12.76	5.32 ± 15.33	1.14 ± 19.09	0.684
Cheeks	T1: Day 30	48	1.56 ± 5.37	−2.43 ± 6.34	3.99 ± 6.80	< 0.001***
	T2: Day 60	48	4.90 ± 7.32	1.35 ± 8.29	3.55 ± 8.64	0.006**
	T3: Day 90	48	13.70 ± 8.20	4.34 ± 8.03	9.36 ± 9.10	< 0.001***
	T4: Day 180	47	9.59 ± 7.59	0.94 ± 7.78	8.65 ± 8.61	< 0.001***
Mouth corners	T1: Day 30	49	2.65 ± 7.06	1.49 ± 9.06	1.16 ± 10.26	0.433
	T2: Day 60	48	4.87 ± 7.83	2.68 ± 12.45	2.19 ± 12.42	0.228
	T3: Day 90	48	12.14 ± 10.74	3.41 ± 13.21	8.73 ± 16.61	0.001**
	T4: Day 180	47	8.24 ± 8.94	2.12 ± 11.91	6.12 ± 14.09	0.005**
Dermal thickness						
Forehead	T1: Day 30	50	4.97 ± 16.79	−2.30 ± 13.49	7.27 ± 19.74	0.019*
	T2: Day 60	49	3.89 ± 15.09	−0.36 ± 15.98	4.25 ± 18.35	0.112
	T3: Day 90	49	4.37 ± 12.53	2.84 ± 14.23	1.53 ± 16.88	0.529
	T4: Day 180	48	5.72 ± 17.39	0.37 ± 12.65	5.35 ± 19.10	0.058
Cheeks	T1: Day 30	50	1.91 ± 7.88	−0.09 ± 11.72	1.99 ± 13.53	0.302
	T2: Day 60	49	1.86 ± 12.11	−0.97 ± 13.99	2.83 ± 15.53	0.208
	T3: Day 90	49	9.71 ± 12.82	1.29 ± 14.15	8.42 ± 14.87	< 0.001***
	T4: Day 180	48	7.61 ± 11.96	1.77 ± 16.16	5.84 ± 18.36	0.032*
Mouth corners	T1: Day 30	50	2.61 ± 19.30	0.59 ± 19.39	2.02 ± 25.47	0.931
	T2: Day 60	49	5.55 ± 22.97	0.88 ± 19.82	4.68 ± 17.84	0.073
	T3: Day 90	49	6.52 ± 16.96	1.35 ± 16.73	5.17 ± 20.89	0.089
	T4: Day 180	48	6.22 ± 18.22	1.34 ± 22.75	4.88 ± 24.87	0.027*
Transepidermal water loss (TEWL)						
Forehead	T1: Day 30	50	−7.66 ± 54.54	−5.31 ± 40.88	−2.36 ± 42.75	0.255
	T2: Day 60	49	−17.30 ± 38.03	−15.36 ± 38.82	−1.94 ± 34.53	0.821
	T3: Day 90	49	8.00 ± 72.29	7.83 ± 64.66	0.17 ± 56.46	0.828
	T4: Day 180	48	3.11 ± 77.29	1.70 ± 68.82	1.41 ± 43.95	0.825
Cheeks	T1: Day 30	50	−7.62 ± 48.21	−12.69 ± 43.69	5.07 ± 48.25	0.772
	T2: Day 60	49	−13.61 ± 71.82	−19.47 ± 47.35	5.86 ± 66.12	0.921
	T3: Day 90	49	−9.70 ± 59.51	−15.21 ± 40.90	5.52 ± 55.41	0.489
	T4: Day 180	46	13.28 ± 98.99	−3.25 ± 61.65	15.29 ± 81.06	0.626
Mouth corners	T1: Day 30	50	−14.79 ± 35.50	−8.91 ± 37.12	−5.89 ± 36.67	0.262
	T2: Day 60	49	−12.57 ± 48.36	−11.13 ± 43.01	−1.43 ± 37.33	0.441
	T3: Day 90	49	−22.13 ± 39.78	−16.14 ± 45.43	−5.99 ± 42.00	0.521
	T4: Day 180	48	−0.36 ± 73.38	−6.05 ± 58.87	5.69 ± 46.74	0.78

Abbreviation: SD, standard deviation.

^a^ *p* Values were derived from paired *t*‐tests or Wilcoxon signed‐rank tests.

* *p* < 0.05.

** *p* < 0.01.

*** *p* < 0.001.

Regarding dermal thickness, A.G.E. cream‐applied side demonstrated thicker dermis than the control side throughout the follow‐up period, regardless of regions of face (Figure 2b,e,h). Statistically, the intervention side showed significant improvement in dermal thickness at forehead on day 30 (Δ = 7.27, *p* = 0.019), at cheeks on day 90 (Δ = 8.42, *p* < 0.001) and day 180 (Δ = 5.84, *p* = 0.032), and at mouth corners on day 180 (Δ = 4.88, *p* = 0.027) (Table 2).

No significant difference in percentage change in TEWL was found throughout the study (Table 2, Figure 2c,f,i) Additionally, we explored the skin hydration level at each follow‐up visit and found A.G.E. cream significantly improved skin hydration level at cheeks. (Appendix, Table S1).

In terms of the overall aesthetic outcome of the combination of MFUS and A.G.E. cream, more patients felt “very satisfied” with the intervention side compared to the control side on day 90 (38.89% vs. 26.53%, *p* = 0.033) and day 180 (45.83% vs. 25.00%, *p* = 0.004), and more dermatologists felt “very satisfied” with the intervention side than the control side on day 90 (24.49% vs. 10.20%). Similarly, regarding wrinkle improvement, skin elasticity improvement, skin plumpness improvement, and skin hydration improvement, there were more proportion of patients and dermatologists who felt “satisfied” or “very satisfied” with the intervention side than the control side at certain follow‐up time points (Table 3).

**TABLE 3:** Patient and dermatologist satisfaction to both sides during follow‐up.

Follow‐up visit	Satisfaction	Patient evaluation			Dermatologist evaluation		
		Intervention	Control	*p* ^a^	Intervention	Control	*p* ^a^
Overall aesthetic outcome							
T1: Day 30	Not satisfied	1 (2.00%)	0 (0%)	0.401	1 (2.00%)	0 (0%)	0.854
	Neutral	3 (6.00%)	4 (8.00%)		13 (26.00%)	15 (30.00%)	
	Satisfied	27 (54.00%)	31 (62.00%)		33 (66.00%)	31 (62.00%)	
	Very satisfied	19 (38.00%)	15 (30.00%)		3 (6.00%)	4 (8.00%)	
T2: Day 60	Not satisfied	1 (2.04%)	0 (0%)	0.437	0 (0%)	0 (0%)	0.852
	Neutral	4 (8.16%)	7 (14.29%)		15 (30.61%)	13 (26.53%)	
	Satisfied	23 (46.94%)	23 (46.94%)		23 (46.94%)	28 (57.14%)	
	Very satisfied	21 (42.86%)	19 (38.78%)		11 (22.45%)	8 (16.33%)	
T3: Day 90	Not satisfied	0 (0%)	0 (0%)	0.033*	0 (0%)	0 (0%)	0.029*
	Neutral	3 (6.12%)	6 (12.24%)		10 (20.41%)	17 (34.69%)	
	Satisfied	27 (55.10%)	30 (61.22%)		27 (55.10%)	27 (55.10%)	
	Very satisfied	19 (38.78%)	13 (26.53%)		12 (24.49%)	5 (10.20%)	
T4: Day 180	Not satisfied	1 (2.08%)	0 (0%)	0.004**	0 (0%)	0 (0%)	0.208
	Neutral	2 (4.17%)	11 (22.92%)		3 (6.25%)	5 (10.42%)	
	Satisfied	23 (47.92%)	25 (52.08%)		37 (77.08%)	38 (79.17%)	
	Very satisfied	22 (45.83%)	12 (25.00%)		8 (16.67%)	5 (10.42%)	
Wrinkle improvement							
T1: Day 30	Not satisfied	1 (2.00%)	3 (6.00%)	0.029*	3 (6.00%)	1 (2.00%)	0.330
	Neutral	9 (18.00%)	13 (26.00%)		13 (26.00%)	23 (46.00%)	
	Satisfied	29 (58.00%)	29 (58.00%)		29 (58.00%)	22 (44.00%)	
	Very satisfied	11 (22.00%)	5 (10.00%)		5 (10.00%)	4 (8.00%)	
T2: Day 60	Not satisfied	0 (0%)	1 (2.04%)	0.020*	4 (8.16%)	2 (4.08%)	0.977
	Neutral	7 (14.29%)	15 (30.61%)		18 (36.73%)	20 (40.82%)	
	Satisfied	31 (63.27%)	22 (44.90%)		18 (36.73%)	21 (42.86%)	
	Very satisfied	11 (22.45%)	11 (22.45%)		9 (18.37%)	6 (12.24%)	
T3: Day 90	Not satisfied	0 (0%)	0 (0%)	0.020*	2 (4.08%)	0 (0%)	0.008**
	Neutral	8 (16.33%)	17 (34.69%)		12 (24.49%)	25 (51.02%)	
	Satisfied	27 (55.10%)	22 (44.90%)		26 (53.06%)	23 (46.94%)	
	Very satisfied	14 (28.57%)	10 (20.41%)		9 (18.37%)	1 (2.04%)	
T4: Day 180	Not satisfied	0 (0%)	1 (2.08%)	< 0.001***	0 (0%)	0 (0%)	0.010*
	Neutral	4 (8.33%)	20 (41.67%)		8 (16.67%)	17 (35.42%)	
	Satisfied	32 (66.67%)	20 (41.67%)		33 (68.75%)	28 (58.33%)	
	Very satisfied	12 (25.00%)	7 (14.58%)		7 (14.58%)	3 (6.25%)	
Skin elasticity improvement							
T1: Day 30	Not satisfied	1 (2.00%)	3 (6.00%)	0.003**	0 (0%)	3 (6.00%)	0.069
	Neutral	6 (12.00%)	15 (30.00%)		17 (34.00%)	22 (44.00%)	
	Satisfied	28 (56.00%)	26 (52.00%)		28 (56.00%)	22 (44.00%)	
	Very satisfied	15 (30.00%)	6 (12.00%)		5 (10.00%)	3 (6.00%)	
T2: Day 60	Not satisfied	0 (0%)	0 (0%)	0.083	0 (0%)	0 (0%)	0.407
	Neutral	8 (16.33%)	16 (32.65%)		13 (27.08%)	17 (34.69%)	
	Satisfied	28 (57.14%)	22 (44.90%)		28 (58.33%)	27 (55.10%)	
	Very satisfied	13 (26.53%)	11 (22.45%)		7 (14.58%)	5 (10.20%)	
T3: Day 90	Not satisfied	0 (0%)	1 (2.04%)	0.005**	1 (2.04%)	0 (0%)	0.221
	Neutral	9 (18.37%)	20 (40.82%)		16 (32.65%)	22 (44.90%)	
	Satisfied	20 (40.82%)	17 (34.69%)		25 (51.02%)	24 (48.98%)	
	Very satisfied	20 (40.82%)	11 (22.45%)		7 (14.29%)	3 (6.12%)	
T4: Day 180	Not satisfied	0 (0%)	2 (4.17%)	< 0.001***	0 (0%)	0 (0%)	0.005**
	Neutral	9 (18.75%)	20 (41.67%)		8 (16.67%)	24 (50.00%)	
	Satisfied	25 (52.08%)	19 (39.58%)		35 (72.92%)	21 (43.75%)	
	Very satisfied	14 (29.17%)	7 (14.58%)		5 (10.42%)	3 (6.25%)	
Skin plumpness improvement							
T1: Day 30	Not satisfied	0 (0%)	2 (4.00%)	< 0.001***	0 (0%)	1 (2.00%)	0.096
	Neutral	5 (10.00%)	18 (36.00%)		24 (48.00%)	28 (56.00%)	
	Satisfied	26 (52.00%)	21 (42.00%)		20 (40.00%)	20 (40.00%)	
	Very satisfied	19 (38.00%)	9 (18.00%)		6 (12.00%)	1 (2.00%)	
T2: Day 60	Not satisfied	0 (0%)	1 (2.04%)	< 0.001***	2 (4.08%)	0 (0%)	0.342
	Neutral	2 (4.08%)	13 (26.53%)		18 (36.73%)	29 (59.18%)	
	Satisfied	28 (57.14%)	24 (48.98%)		24 (48.98%)	16 (32.65%)	
	Very satisfied	19 (38.78%)	11 (22.45%)		5 (10.20%)	4 (8.16%)	
T3: Day 90	Not satisfied	0 (0%)	0 (0%)	0.045*	2 (4.08%)	1 (2.04%)	0.036*
	Neutral	7 (14.29%)	16 (32.65%)		16 (32.65%)	28 (57.14%)	
	Satisfied	24 (48.98%)	20 (40.82%)		25 (51.02%)	19 (38.78%)	
	Very satisfied	18 (36.73%)	13 (26.53%)		6 (12.24%)	1 (2.04%)	
T4: Day 180	Not satisfied	0 (0%)	1 (2.08%)	< 0.001***	0 (0%)	0 (0%)	0.003**
	Neutral	6 (12.50%)	22 (45.83%)		13 (27.08%)	31 (64.58%)	
	Satisfied	27 (56.25%)	15 (31.25%)		30 (62.50%)	14 (29.17%)	
	Very satisfied	15 (31.25%)	10 (20.83%)		5 (10.42%)	3 (6.25%)	
Skin hydration improvement							
T1: Day 30	Not satisfied	1 (2.00%)	0 (0%)	0.153	0 (0%)	0 (0%)	0.256
	Neutral	8 (16.00%)	14 (28.00%)		14 (28.00%)	17 (34.00%)	
	Satisfied	28 (56.00%)	27 (54.00%)		26 (52.00%)	27 (54.00%)	
	Very satisfied	13 (26.00%)	9 (18.00%)		10 (20.00%)	6 (12.00%)	
T2: Day 60	Not satisfied	1 (2.04%)	0 (0%)	0.016*	1 (2.04%)	0 (0%)	0.976
	Neutral	3 (6.12%)	17 (34.69%)		14 (28.57%)	15 (30.61%)	
	Satisfied	31 (63.27%)	19 (38.78%)		24 (48.98%)	26 (53.06%)	
	Very satisfied	14 (28.57%)	13 (26.53%)		10 (20.41%)	8 (16.33%)	
T3: Day 90	Not satisfied	0 (0%)	0 (0%)	0.001**	0 (0%)	0 (0%)	0.131
	Neutral	5 (10.20%)	18 (36.73%)		12 (24.49%)	18 (36.73%)	
	Satisfied	22 (44.90%)	17 (34.69%)		32 (65.31%)	29 (59.18%)	
	Very satisfied	22 (44.90%)	14 (28.57%)		5 (10.20%)	2 (4.08%)	
T4: Day 180	Not satisfied	1 (2.08%)	3 (6.25%)	0.001**	0 (0%)	0 (0%)	0.097
	Neutral	10 (20.83%)	16 (33.33%)		12 (25.00%)	16 (34.04%)	
	Satisfied	18 (37.50%)	21 (43.75%)		28 (58.33%)	28 (59.57%)	
	Very satisfied	19 (39.58%)	8 (16.67%)		8 (16.67%)	3 (6.38%)	

* *p* < 0.05.

** *p* < 0.01.

*** *p* < 0.001.

Regarding fine lines measured by Griffith's scale, the study found significantly greater improvement from baseline at the mouth corner at the intervention side on day 180, compared with the control side (−1.38 vs. −1.10, *p* = 0.035). There were no significant differences between the two sides at other time points or other regions of the face.

**FIGURE 3:**
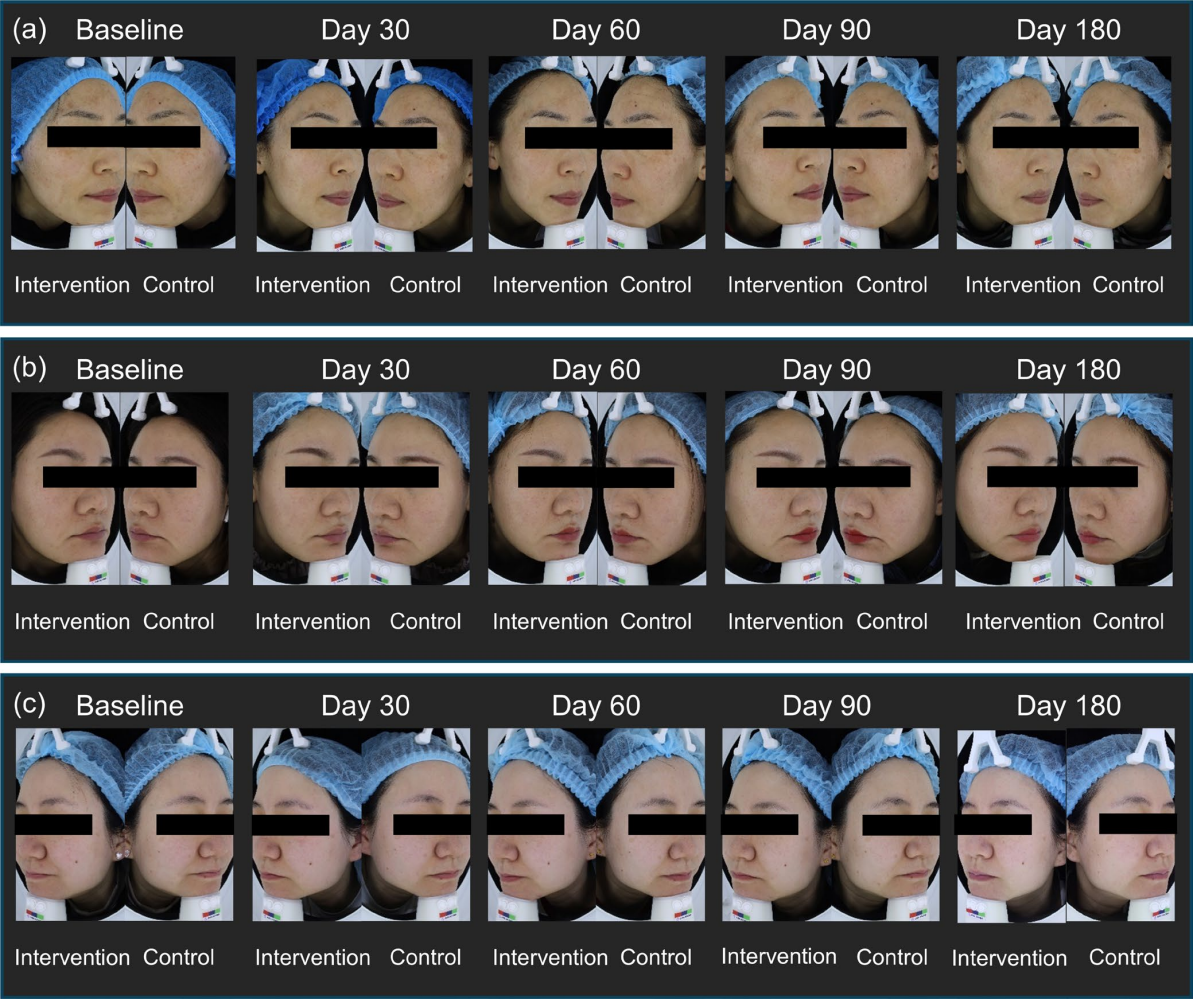
Clinical photographs of both sides of the facial skin at baseline and during follow‐up. (a) Female, 36 years, with mild‐to‐moderate skin laxity and dryness, enlarged pores, obvious wrinkles at mouth corners, and a few fine lines on the cheeks and forehead; (b) Female, 34 years, with mild‐to‐moderate skin laxity and dryness, localized adiposities, enlarged pores, obvious wrinkles at mouth corners, and a few fine lines on the cheeks; (c) Female, 37 years, with skin dryness, localized adiposities, enlarged pores, and fine lines at eye corners, mouth corners, and cheeks. Intervention: A.G.E. cream; Control: Standard moisturizer.

Clinical photographs of three patients at baseline and during follow‐up were shown in Figure 3a–c.

### 3.4 Safety

On the second day following MFUS treatment, one patient reported expansive erythema and a few blisters on both sides of the cheeks. These symptoms persisted for approximately 7 days. The patient discontinued the use of the A.G.E. cream on the day the symptoms appeared and resumed use after a week. Following the resumption of the A.G.E. cream, no additional adverse events were reported.

## 4 Discussion

This was the first randomized controlled trial in China investigating the efficacy and satisfaction of the combination of MFUS and functional skincare product (A.G.E. cream) containing blueberry extract and Pro‐xylane for antiaging.

As an effective energy modality for antiaging, MFUS provides dermal and subdermal heating to induce collagen denaturation and remodeling, leading to skin tightening and rejuvenation which can last for approximately 3 months [25, 26]. To enhance and prolong the clinical response of MFUS has become an unmet demand for patients and dermatologists. Expert consensus on the clinical practice of integrated skincare released in China recommended combining clinically proven functional skincare products with cosmetic procedures to achieve synergistic and prolonged effects, mitigate side effects, and improve patient satisfaction [27]. Our study showed the combination of A.G.E. cream and MFUS demonstrated significant improvements in aesthetic outcomes compared to the standard moisturizer on Day 90 and Day 180 regarding GAIS evaluated by investigators. As a result, this study was a promising attempt under this integrative skincare concept.

In terms of skin parameters, our study found a notable improvement in skin elasticity and dermal thickness, as well as higher patient and dermatologist satisfaction with skin elasticity and plumpness improvement with the use of A.G.E. cream, which can be attributed to its key ingredient, Pro‐Xylane. Pro‐Xylane, also known as C‐xyloside, is a sugar molecule extracted from beech wood. Evidence has shown that C‐xyloside can significantly increase the deposition of basement membrane and dermal–epidermal junction proteins in a reconstructed skin model, as well as boost collagen VII gene expression [28]. By promoting the production of proteoglycans, it can aid in reconstructing the skin's extracellular matrix, leading to enhanced skin elasticity and thickness [29]. Blueberry extract is another key ingredient in the A.G.E. cream. Blueberries are rich in bioactive compounds such as flavonoids and polyphenols, which possess strong antioxidant, anti‐inflammatory, and anti‐glycation properties, indicating that blueberries might be an effective topical defense to common environmental insults [15, 30]. A 12‐week clinical trial involving the application of blueberry extract on the hands, arms, and faces of women with type II diabetes demonstrated significant improvements in skin wrinkle reduction, smoothness, and hydration [23]. Our research also noted comparable outcomes in enhancing skin hydration and satisfaction with wrinkle improvement among patients and dermatologists.

An important aspect of our study design was the evaluation of skin parameters across various facial regions, including the forehead, cheeks, and mouth corners. Our study observed varying degrees of improvement at the A.G.E. cream‐applied side in these different facial regions. For example, skin elasticity showed a more pronounced enhancement in the cheeks and mouth corners, with no significant improvement noted in the forehead region. Previous research has indicated that the aging process differs among various body parts. In the context of facial aging patterns, a study on Chinese females revealed that the eyes exhibited the fastest aging rate between 19 and 32 years, followed by the cheeks and mouth corners, and then the forehead [3]. This indicated that A.G.E. cream might be able to provide targeted improvement in specific facial regions such as the cheeks and mouth corners, where aging may be more pronounced, and thus catering to the specific needs of patients by addressing these areas of concern effectively.

In addition to objective skin parameter evaluations, our study also incorporated subjective measures from patients and dermatologists to assess their satisfaction level for the combined use of A.G.E. cream following MFUS. The use of such patient‐reported measures in facial aesthetic assessment has been widely acknowledged for its ability to accommodate patients with varying motivations or desired results. This study revealed a significantly higher patient satisfaction rate in A.G.E. creams than in the standard moisturizer, in terms of all assessment questions including overall aesthetic outcome, wrinkle improvement, skin elasticity improvement, skin plumpness improvement, and skin hydration improvement. This underscored the effectiveness of the A.G.E. cream combined with MFUS in meeting the diverse needs and expectations of patients for facial antiaging and rejuvenation.

This study also has its limitations. One limitation of this study was that patients were not blinded, which could introduce bias when assessing satisfaction levels with the use of A.G.E. cream following MFUS. Another limitation was the inability to ensure adherence and consistency of application since the cream was self‐administered by the patients. Although certain measures, such as regular reminders by study staff and patient diaries, were implemented, these may not have fully mitigated the issue. Lastly, the study population was predominantly female and relatively younger. The imbalance could be due to the higher willingness of females with a relatively younger age to participate in skincare studies. Future studies should include participants with more diverse demographic characteristics to enhance the generalizability of the findings.

## 5 Conclusion

Findings in this study demonstrated that the application of a topical product containing blueberry extract and Pro‐xylane (A.G.E. cream) following the MFUS treatment could significantly improve skin elasticity, dermal thickness, fine lines, and patient as well as dermatologist satisfaction. This study is a promising attempt to combine functional skincare products with MFUS to consolidate and prolong the antiaging effects.

## Author Contributions

Xiaoling Jiang and Weiming Song led the investigation. Shiqi Peng, Ying Chen, and Yuqing Nan supported the investigation. All authors equally contributed to the conceptualization. Xiaoling Jiang was responsible for project administration.

## Ethics Statement

This study has been approved by the Ethics Committee of YESSKIN Clinic on December 12th, 2022. The approval number is 20221202. Written informed consent was obtained from each subject. Participants have permitted the use of photographs through photo consent forms.

## Conflicts of Interest

L'Oreal Dermatological Beauty China, SkinCeuticals provided funding and study materials (A.G.E. Interrupter Cream) for the trial. There were no other grants or funding to be disclosed by authors Xiaoling Jiang, Shiqi Peng, Ying Chen, Yuqing Nan, or Weimin Song.

## Supporting information


**Table S1.** Percentage changes from baseline in skin hydration at both sides during follow‐up.

## Data Availability

The data that support the findings of this study are available on request from the corresponding author. The data are not publicly available due to privacy or ethical restrictions.
