# Supplementary material for: A Randomized, Investigator‐Blinded, Split‐Face, Controlled Trial Evaluating the Efficacy and Satisfaction of a Topical Product Containing Blueberry Extract and Pro‐Xylane Combined With Micro‐Focused Ultrasound for Anti‐aging
Source: J Cosmet Dermatol. 2025 Jul 10;24(7):e70281. doi: 10.1111/jocd.70281 (PMC12242364; doi:10.1111/jocd.70281)
Supplement: Supplementary file 1 — Table S1. Percentage changes from baseline in skin hydration at both sides during follow‐up. [file JOCD-24-e70281-s001.docx]

**Table S1 Percentage changes from baseline in skin hydartion at both sides during follow-up**

| Skin hydration | Follow-up time points | N | Mean percentage change from baseline, mean ± SD | | Difference between groups (Δ), mean ± SD | *p – value* |
| --- | --- | --- | --- | --- | --- | --- |
|  |  |  | Intervention | Control |  |  |
| Forehead | T1: Day 30 | 50 | 7.69 ± 35.80 | 8.85 ± 35.94 | -1.16 ± 26.34 | 0.931 |
|  | T2: Day 60 | 49 | 16.26 ± 43.02 | 16.02 ± 35.70 | 0.24 ± 28.21 | 0.954 |
|  | T3: Day 90 | 49 | 18.51 ± 53.83 | 18.97 ± 43.87 | -0.46 ± 27.36 | 0.396 |
|  | T4: Day 180 | 48 | 2.00 ± 31.28 | -0.06 ± 29.24 | 2.06 ± 23.74 | 0.551 |
| Cheeks | T1: Day 30 | 50 | 8.79 ± 23.03 | 2.20 ± 18.32 | 6.59 ± 19.16 | 0.011^*^ |
|  | T2: Day 60 | 49 | 14.92 ± 28.00 | 5.19 ± 21.58 | 9.73 ± 23.12 | 0.015^*^ |
|  | T3: Day 90 | 49 | 17.98 ± 29.72 | 5.40 ± 23.06 | 12.58 ± 18.21 | <0.001^***^ |
|  | T4: Day 180 | 48 | 5.42 ± 19.48 | 3.30 ± 18.45 | 2.12 ± 14.81 | 0.326 |
| Mouth corners | T1: Day 30 | 50 | 3.38 ± 21.27 | 0.37 ± 17.91 | 3.01 ± 14.89 | 0.125 |
|  | T2: Day 60 | 49 | 10.29 ± 27.29 | 2.24 ± 23.35 | 8.04 ± 23.94 | 0.08 |
|  | T3: Day 90 | 49 | 10.43 ± 27.44 | 1.83 ± 23.77 | 8.60 ± 19.28 | 0.003^**^ |
|  | T4: Day 180 | 48 | 0.30 ± 17.05 | 0.48 ± 18.38 | -0.19 ± 18.83 | 0.946 |

^+^*p*-values were derived from paired t-tests or Wilcoxon signed-rank tests.

^*^: *p*<0.05; ^**^: *p*<0.01; ^***^: *p*<0.001
